# Underestimation of pregnancy risk among women in Vietnam

**DOI:** 10.1186/s12905-020-01013-6

**Published:** 2020-07-29

**Authors:** Jessica Londeree, Nghia Nguyen, Linh H. Nguyen, Dung H. Tran, Maria F. Gallo

**Affiliations:** 1 Division of Epidemiology, The Ohio State University, College of Public Health, Cunz Hall, 1841 Neil Avenue, Columbus, OH 43210 USA; 2 Department of Obstetrics and Gynecology, Vinmec International Hospital, 458 Minh Khai, Hanoi, Vietnam; 3 Department of Research and Training, Hanoi Obstetrics and Gynecology Hospital, La Thanh Street, Hanoi, Vietnam

**Keywords:** Contraception, Health knowledge, attitudes, practice, Pregnancy, unplanned, Risk assessment, Vietnam

## Abstract

**Background:**

Addressing women’s inaccurate perceptions of their risk of pregnancy is crucial to improve contraceptive uptake and adherence. Few studies, though, have evaluated the factors associated with underestimation of pregnancy risk among women at risk of unintended pregnancy.

**Methods:**

We assessed the association between demographic and behavioral characteristics and underestimating pregnancy risk among reproductive-age, sexually-active women in Hanoi, Vietnam who did not desire pregnancy and yet were not using highly-effective contraception (*N* = 237). We dichotomized women into those who underestimated pregnancy likelihood (i.e., ‘very unlikely’ they would become pregnant in the next year), and those who did not underestimate pregnancy likelihood (i.e., ‘somewhat unlikely,’ ‘somewhat likely’ or ‘very likely’). We used bivariable and multivariable logistic regression models to identify correlates of underestimating pregnancy risk.

**Results:**

Overall, 67.9% (*n* = 166) of women underestimated their pregnancy risk. In bivariable analysis, underestimation of pregnancy risk was greater among women who were older (> 30 years), who lived in a town or rural area, and who reported that it was “very important” or “important” to them to not become pregnant in the next year. In multivariable analysis, importance of avoiding pregnancy was the sole factor that remained statistically significantly associated with underestimating pregnancy risk (odds ratio [OR]: 0.11; 95% confidence interval [CI], 0.05–0.25). In contrast, pregnancy risk underestimation did appear to vary by marital status, ethnicity, education or other behaviors and beliefs relating to contraceptive use.

**Conclusions:**

Findings reinforce the need to address inaccurate perceptions of pregnancy risk among women at risk of experiencing an unintended pregnancy.

## Background

Of pregnancies occurring worldwide from 2000 to 2014, an estimated 44% of were unintended [1]. Unintended pregnancies, defined as pregnancies that are unwanted or mistimed at the time of conception, pose a substantial social and economic burden for women and their families. Consequences of these pregnancies include poor birth outcomes [2], increased levels of pregnancy-related morbidity and mortality [3, 4], as well as mental health concerns and lost educational opportunities among children [5, 6]. Despite these consequences, a large gap remains between the availability of contraceptive methods and their use. An estimated 80% of the 85 million women annually who have an unintended pregnancy are not using contraception at the time of conception [4]. In lower and middle-income countries, where most unintended pregnancies occur [1, 4], and where the health infrastructure is often ill-equipped to handle the consequences of unintended pregnancy, understanding the barriers to contraception use among women who desire to prevent pregnancy is critical.

According to the health belief model, appropriate perception of susceptibility to a given health outcome is a key determinant of health behavior and behavior change [7, 8]. A woman’s cognizance of her risk of unintended pregnancy then may play a crucial role in contraceptive behavior and adherence. Indeed, underestimation of pregnancy risk has been found to lead to unmet contraceptive need [9, 10] and, subsequently, unintended pregnancy [11, 12].

Several studies across a range of settings have revealed a significant discrepancy between perceived and actual pregnancy risk. In a study among reproductive-age women in France, Moreau and Bohet found that, among women who reported inconsistent use of contraception or unprotected intercourse in the last 4 weeks, 63% did not think they could become pregnant unintentionally [13]. Sinai et al. observed that, among women in Mali and Benin, 33.7% of women at risk of pregnancy (i.e., women who were fecund and sexually active) believed that they could not become pregnant [14]. In another study of women attending reproductive healthcare clinics in the United States, Biggs et al. found that 27% of women planning to use no method or a low-efficacy contraceptive method (i.e., natural family planning, withdrawal, diaphragm, or sponge) underestimated their risk of pregnancy from engaging in 1 year of unprotected intercourse [15].

Although addressing inaccurate perceptions of pregnancy risk may be central to preventing unintended pregnancy, few studies to date have evaluated the factors associated with underestimation of pregnancy risk among women at risk. Assessing these factors could help identify target populations for interventions to address the gap between perceived and actual pregnancy risk and, accordingly, the gap between the existence of effective contraception and its use. The aim of the present study was then to assess the prevalence and correlates of underestimation of pregnancy risk among sexually- active women in Hanoi, Vietnam, who were not using a highly-effective method of contraception and yet did not desire pregnancy.

## Methods

We analyzed data from cross-sectional, convenience study of women in Hanoi, Vietnam. The parent study’s primary objective was to assess a method of measuring beliefs concerning contraception safety and naturalness, and these findings will be reported elsewhere. The parent study enrolled 500 adult women of reproductive age (18–45 years) attending the obstetrics-gynecology department of a large public hospital for routine care or accompanying someone at the facility during November 2017 to September 2018. To participate in the study, women had to have at least a minimal level of literacy, report being comfortable using a computer, be sexually active (defined as ≥1 penile-vaginal act in past month), not be pregnant or breastfeeding, and not want a pregnancy within the next 12 months. Written consent was provided by participants before enrollment, and the research was approved by institutional review boards at The Ohio State University and the Hanoi School of Public Health.

**Fig. 1:**
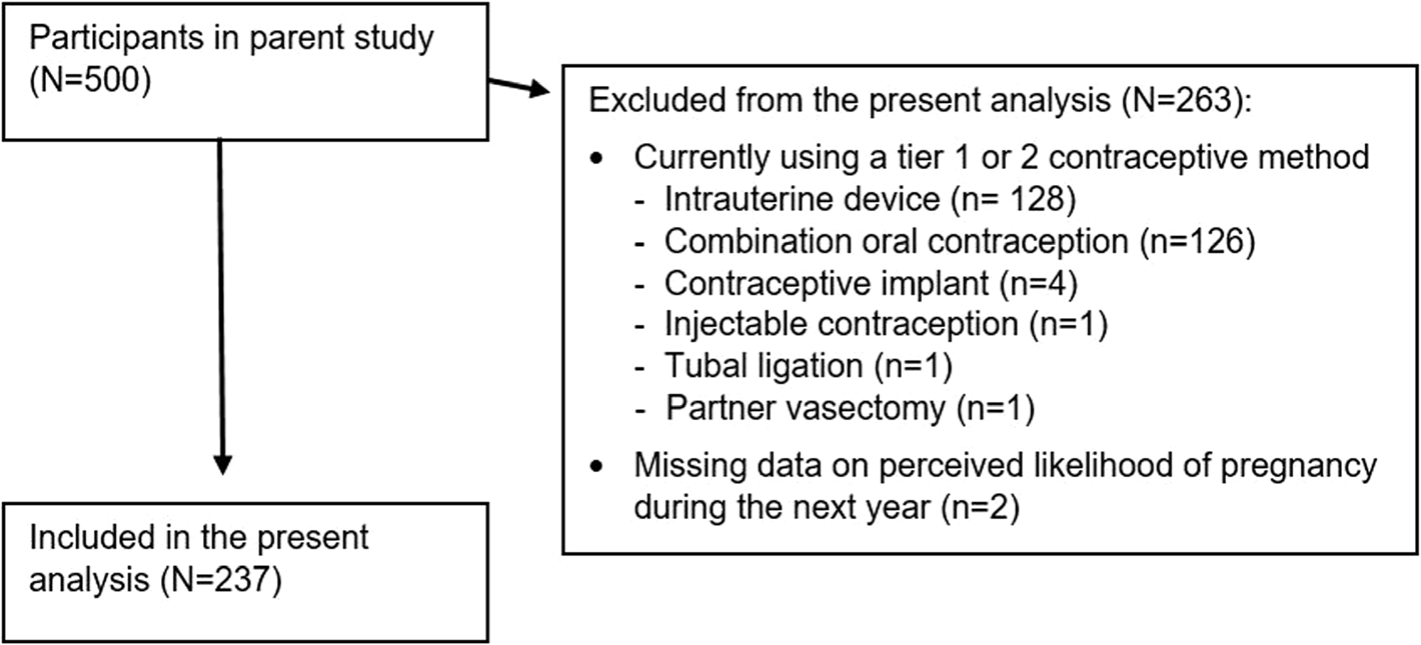
Participant disposition

We administered a questionnaire on demographics and contraception-related beliefs and behaviors. As part of this questionnaire, we asked participants to report the likelihood (“very unlikely, somewhat unlikely, somewhat likely and very likely”) they would become pregnant in the next year. For the present study, we restricted our analysis to women who were not currently using a highly effective method of contraception, specifically either a tier 1 (i.e., implant, intrauterine device, tubal ligation or vasectomy) or a tier 2 method (i.e., injectable contraception, lactational amenorrhea, oral contraception, patch or vaginal ring) [16]. Thus, we excluded 261 women who were using a tier 1 or 2 method and 2 women who were missing data on perceived likelihood of pregnancy over the next year (Fig. 1).

Based on responses regarding the perceived likelihood of pregnancy, we dichotomized women into those who underestimated pregnancy likelihood (i.e., women who reported it was ‘very unlikely’ they would become pregnant in the next year), and those who did not underestimate pregnancy likelihood (i.e., women who reported it was ‘somewhat unlikely,’ ‘somewhat likely,’ or ‘very likely’ they would become pregnant in the next year). Based on the literature, we selected the following demographic characteristics to evaluate as potential correlates of pregnancy likelihood underestimation [13, 15, 17]: age (categorized into 21–31 years, 32–36 years, and 37–45 years); residence (city vs. town or rural area); marital status (married vs. other); ethnicity (Kinh vs. other); education (secondary or lower vs. higher); and monthly household income (< 15 million Vietnamese dong [equivalent to ~ 650 U.S. dollars] vs. higher). We also assessed the following contraception-related beliefs and behaviors: current use of male condoms (yes vs. no), current use of traditional contraceptive methods (i.e., rhythm, withdrawal; yes vs. no), ever been pregnant (yes vs. no), experience ever discussing contraceptive methods with health provider (yes vs. no), and ambiguity towards becoming pregnant (ambiguous vs. not-ambiguous). In response to the question “How important is it to you to not become pregnant in the next year?” women who reported it was ‘very important’ or ‘important’ were categorized as not-ambiguous toward becoming pregnant, while those who reported it was ‘neutral’ or ‘not important’ were categorized as ambiguous towards becoming pregnant.

In separate bivariable logistic regression models, we assessed the relationship between potential correlates and pregnancy risk underestimation. We then ran a multivariable logistic regression model fitted with all correlates that were associated with the outcome in the bivariable analysis using a *p*-value of < 0.25 [18]. We used SAS 9.4 (SAS, Cary, NC) for all analyses.

## Results

The analysis is based on 237 women who were susceptible to unintended pregnancy (i.e., sexually-active, reproductive-age women who were not using a tier 1 or 2 method of contraception and did not wish to become pregnant in the next year). Most participants resided in a city (88.2%), had attended education beyond upper secondary school (73.0%), were married (93.7%), were ethnically Kinh (93.7%), and reported a household income of > 15 million Vietnamese dong (71.7%) (Table 1). The median age of participants was 34.1 years (standard deviation, 5.3; range, 21–45 years). Participants reported the following methods of contraception (based on a hierarchical categorization, in which those reporting multiple methods only had their first response in the following ordered list included): male condom (*n* = 166), female condom (*n* = 7), withdrawal (*n* = 53), rhythm/periodic abstinence (*n* = 4) or no method (*n* = 9). Overall, 67.9% of women believed it was very unlikely that they would become pregnant, while 9.7% believed it was somewhat unlikely, 17.2% believed it was somewhat likely and 2.9% believed it was very likely.

**Table 1:** Demographic and behavioral characteristics of women at risk of unintended pregnancy^a^ in Hanoi, Vietnam by perceived pregnancy risk (*N* = 237)

		Perceived pregnancy risk	
	Overall	Underestimated (*n* = 166)	Not underestimated (*n* = 71)
	n (%)	n (%)	n (%)
Age in years			
21–31	87 (36.7)	48 (29.0)	39 (54.9)
32–36	74 (31.2)	59 (35.5)	15 (21.1)
37–45	76 (32.1)	59 (35.5)	17 (24.0)
Residence			
Town or rural area	28 (11.8)	25 (15.1)	3 (4.2)
City	209 (88.2)	141 (84.9)	68 (95.8)
Highest level of education completed			
Upper secondary or less	64 (27.0)	49 (29.5)	15 (21.1)
Higher	173 (73.0)	117 (70.5)	56 (78.9)
Marital status			
Married	222 (93.7)	157 (94.6)	65 (91.5)
Other	15 (6.3)	9 (5.4)	6 (8.5)
Ever been pregnant			
Yes	219 (92.4)	161 (97.0)	58 (81.7)
No	4 (1.7)	1 (0.6)	3 (4.2)
Missing	14 (5.9)	4 (2.4)	10 (14.1)
Ethnicity			
Kinh	222 (93.7)	156 (94.0)	66 (93.0)
Non-Kinh	15 (6.3)	10 (6.0)	5 (7.0)
Monthly household income			
≥ 15,000,000 Vietnamese dong	170 (71.7)	117 (70.5)	53 (74.6)
< 15,000,000 Vietnamese dong	44 (18.6)	30 (18.1)	14 (19.7)
Missing	23 (9.7)	19 (11.4)	4 (5.6)
Current male condom use			
Yes	165 (69.6)	117 (70.5)	48 (67.6)
No	72 (30.4)	49 (29.5)	23 (32.4)
Current traditional contraception use^b^			
Yes	147 (62.0)	100 (60.2)	47 (66.2)
No	90 (38.0)	66 (39.8)	24 (33.8)
Frequency of sexual intercourse			
At least once per week	187 (78.9)	131 (78.9)	56 (78.9)
Less than once per week	41 (17.3)	31 (18.7)	10 (14.1)
Missing	9 (3.8)	4 (2.4)	5 (7.0)
Health provider discussed contraception			
Yes	126 (53.2)	83 (50.0)	43 (60.6)
No	110 (46.4)	82 (49.4)	28 (39.4)
Missing	1 (0.4)	1 (0.6)	0 (0)
Pregnancy ambivalence			
Ambivalent	49 (20.7)	16 (9.6)	33 (46.5)
Not-ambivalent	187 (78.9)	150 (90.4)	37 (52.1)
Missing	1 (0.4)	0 (0)	1 (1.4)

^a^Women were classified as at risk of unintended pregnancy if they were sexually-active, of reproductive-age, did not desire to become pregnant and were not using a highly-effective contraceptive method

^b^Traditional contraception included use of rhythm and withdrawal

In bivariable analysis, age, residence and pregnancy ambivalence were statistically significantly associated with pregnancy risk underestimation (Table 2). Compared to women in the younger age group (21–31 years), women ages 32–36 years and 37–45 years had 3.2 (95% confidence interval [CI], 1.6–6.5) and 2.8 (95% CI, 1.4–5.6) times the odds of pregnancy risk underestimation, respectively (Table 2). Women living in a town or rural area had four-fold greater odds of pregnancy risk underestimation relative to women living in a city (OR, 4.0; 95% CI, 1.0–15.9). Compared to women who were not ambivalent about becoming pregnant, women who were ambivalent about pregnancy had lower odds of pregnancy risk underestimation (OR: 0.12; 95% CI, 0.1–0.2). Women who underestimated their pregnancy risk did not differ significantly from women who did not underestimate their risk by marital status, ethnicity, income, reported use of condoms or traditional contraceptive methods (i.e., rhythm or withdrawal), frequency of sexual intercourse or by experience with health provider discussing contraception use.

**Table 2:** Bivariable and multivariable analyses of correlates of pregnancy risk underestimation among women at risk of unintended pregnancy ^a^ in Hanoi, Vietnam (*N* = 237)

	OR	(95% CI)	aOR^b^	(95% CI)
Age in years				
21–31	Ref	**–**	Ref	**–**
32–36	3.20	(1.58–6.48) ^c^	2.21	(0.95–5.16)
37–45	2.82	(1.42–5.60) ^c^	1.95	(0.86–4.45)
Residence				
Town or rural area	4.02	(1.17–13.78) ^c^	3.91	(0.96–13.78)
City	Ref	–	Ref	–
Highest level of education completed				
Upper secondary or less	1.56	(0.81–3.03) ^c^	1.37	(0.61–3.08)
Higher	Ref	–	Ref	–
Marital status				
Married	1.61	(0.55–4.71)	–	–
Other	Ref	–	–	–
Ever been pregnant				
Yes	8.33	(0.85–81.66) ^c^	5.76	(0.50–65.83)
No	Ref	–	Ref	–
Ethnicity				
Kinh	1.18	(0.39–3.59)	–	–
Non-Kinh	Ref	–	–	–
Monthly household income				
≥ 15,000,000 Vietnamese dong	0.97	(0.48–1.98)	–	–
< 15,000,000 Vietnamese dong	Ref	–	–	–
Current male condom use				
Yes	1.14	(0.63–2.08)	–	–
No	Ref	–	–	–
Current traditional contraception use^d^				
Yes	0.77	(0.43–1.38)	–	–
No	Ref	–	–	–
Frequency of sexual intercourse				
At least once per week	0.75	(0.35–1.64)	–	–
Less than once per week	Ref	–	–	–
Health provider discussed contraception				
Yes	0.65	(0.37–1.16) ^c^	0.71	(0.35–1.43)
No	Ref	–	Ref	–
Pregnancy ambivalence				
Ambivalent	0.12	(0.06–0.24) ^c^	0.11	(0.05–0.25)
Not-ambivalent	Ref	–	Ref	–

*OR* Odds Ratio, *CI* Confidence Interval, *aOR* Adjusted Odds Ratio

^a^ Women were classified as at risk of unintended pregnancy if they were sexually-active, of reproductive-age, did not desire to become pregnant and were not using a highly-effective contraceptive method

^b^ Adjusted for all variables in column

^c^*P*-value < 0.25 and thus was included in the initial full model for the multivariable analysis

^d^ Traditional contraception included use of rhythm and withdrawal

In multivariable analysis, which was fit with variables associated with pregnancy risk underestimation at *p* < 0.25 in bivariate analysis, only pregnancy ambiguity remained statistically significantly associated with pregnancy risk underestimation (aOR: 0.11; 95% CI, 0.05–0.25; Table 2). Age and residence within a town or rural area were associated with greater pregnancy risk underestimation; however, this association was not significant at alpha = 0.05 level.

## Discussion

Underestimation of pregnancy risk was prevalent among this population of women at risk of unintended pregnancy in Vietnam, with most women (67.9%) perceiving it to be ‘very unlikely’ they could become pregnant. Risk underestimation was greater among women who were older, among women who lived in town or rural areas and among women who were not ambivalent about becoming pregnant in the next year (i.e., perceived avoiding pregnancy as important or very important). Pregnancy risk underestimation did not appear to vary, though, by marital status, ethnicity, education or other behaviors and beliefs relating to contraceptive use.

We restricted our study population to women who were sexually-active (defined as at least one act in the past month) and not currently using highly-effective contraception. Women having less frequent sex (including those who experience forced sex) or those using a highly-effective contraceptive method can face the risk unintended pregnancy. However, we focused our study on assessing correlates of reported unintended pregnancy risk among women who are most susceptible to unintended pregnancy, and thus should be the target of public health interventions. As perceived susceptibility to a health outcome is a key determinant of behavior change, the high prevalence of pregnancy risk underestimation in our sample reinforces the need for interventions to address inaccurate perceptions of pregnancy risk, especially among women who are older and who live in non-urban settings. Future studies should assess the effect of interventions shown to improve reproductive and contraceptive knowledge, such as entertainment education (e.g., radio drama) [19], tailored oral education [20], or other health promotion materials (e.g., posters, brochures) [21, 22], on correcting inaccurate perceptions of pregnancy risk in this setting.

Our study also reinforces the need for a more nuanced categorization of contraceptive need. At present, contraceptive use is commonly categorized into ‘met’ and ‘unmet’ need, based on fecundity, sexual activity and current contraceptive use. Incorporating perception of contraceptive need into the categorization of contraceptive use could further elucidate why some women fail to use effective contraceptive methods, despite their availability. One such strategy of categorization, known as the Tékponon Jikuagou approach, splits contraceptive use into five categories: real met need (current users of a modern method), perceived met need (current users of a traditional method), real no need, perceived no need (those with a physiological need for family planning who perceive no need), and perceived unmet need (those who realize they have a need but do not use a method) [14]. The use of this categorization could better inform targeted behavioral interventions to prevent unintended pregnancy.

We note that our results should not be generalized to users of highly-effective contraception as such generalizations could be subject to selection bias. Our findings concerning pregnancy ambivalence illustrate this potential source of bias; the women in our sample who were not ambiguous about avoiding pregnancy were more likely to underestimate the probability they would become pregnant. Initially, this finding may seem unusual as one may expect that those who are most adamant about avoiding pregnancy would be more aware of their pregnancy risk. However, we may also expect that most women who choose not to use contraceptives, despite having great desire to avoid pregnancy, would believe it is improbable they could naturally conceive. In short, by selecting on non-use of highly-effective contraception, we observe an association between pregnancy ambiguity and risk underestimation that may otherwise not be observed in a sample of all women of reproductive age. Indeed, in a study of contraceptive users and non-users in the United States, Rahman et al. found that women who were ambivalent about pregnancy were *more* likely to have accurate perceptions of their risk of pregnancy [17], in contrast to our own findings.

Regarding the association between pregnancy risk underestimation and age, we acknowledge that the ability to become pregnant naturally declines with increasing age. Women’s fecundity begins to gradually lessen at age 32, before dropping rapidly at the age of 37 with the onset of perimenopausal menstrual irregularity [23]. Thus, though all women in this sample were of reproductive age, the low perceived pregnancy likelihood among older women could be based – in part – on biologic reality. Nonetheless, if reproductive age women are sexually active and not using highly-effective contraception, the risk of unintended pregnancy persists. Therefore, the belief that pregnancy is very unlikely remains an underestimation of pregnancy likelihood, especially for women under the age of 37 years. Additionally, we observed that the level of pregnancy risk underestimation was similar in older age groups: women ages 32–37 years had nearly identical odds of pregnancy risk underestimation relative to women ages 38–45 years. These findings further suggest that women’s perception of their ability to become pregnant does not fully align with the natural decline in fecundity with age.

Our study population was relatively homogenous; most participants in our sample were married, of the Kinh ethnicity and resided in an urban setting. This homogeneity limits the generalizeability of our findings to similar population groups. Additionally, our convenience sampling strategy furthers limits the generalizability of our findings, as women seeking care from a single facility in Hanoi may not be representative of all reproductive-age women in the region. Despite these limitations, our study is the first to quantitatively assess correlates of inaccurate pregnancy risk estimation in a non-Western context.

## Conclusions

As women in middle and low-income countries experience disproportionate rates of unintended pregnancy [1], it is crucial to assess potential modifiable factors contributing to poor contraceptive use in these regions. In the present study of women at risk of unintended pregnancy in Hanoi, Vietnam, most women underestimated their risk of pregnancy. These findings highlight the need for interventions to address women’s misconceptions of their pregnancy risk.

## Data Availability

The study dataset is available from the corresponding author following institutional approvals.

## Abbreviations

aOR: adjusted odds ratio
CI: Confidence interval
OR: Odds ratio
